# Endovascular treatment for mycotic aneurysm using pyoktanin- applied devices

**DOI:** 10.1186/s42155-020-00151-0

**Published:** 2020-11-08

**Authors:** Kei Kazuno, Hajime Kinoshita, Mariko Hori, Takamichi Yosizaki, Atsusi Tamura, Hiroshige Sato, Seiichiro Murata

**Affiliations:** 1 Department of Cardiovascular Surgery, Itabashi Chuo Medical Center, 2-12-7 Azusawa Itabashi-ku, Tokyo, 174-0051 Japan; 2 Department of Cardiovascular Surgery, Tokushima Prefectural Central Hospital, 1-10-3 Kuramoto-cho Tokushima-city, Tokushima, 770-8539 Japan

**Keywords:** Aneurysm, Methylrosanilide chloride, Endovascular procedures, Thoracic surgery

## Abstract

**Background:**

Mycotic thoracic aortic aneurysm is an extremely rare but serious disease because it can easily rupture and has a high mortality rate. The standard therapy for it comprises graft replacement and debridement using systemic antibiotics; nonetheless, this has a high mortality rate and complications. Endovascular aortic repair is considered a bridging therapy before open surgery. However, we have used it at our institution for the radical treatment of mycotic thoracic aortic aneurysm utilizing pyoktanin (methylrosanilide chloride)-applied devices. Thus, the aim of this study was to report our clinical experience with pyoktanin-applied thoracic endovascular aortic repair for the treatment of mycotic thoracic aortic aneurysm, including its effects.

**Methods:**

From April 2017 to July 2019, we performed thoracic endovascular aortic repair using pyoktanin for eight cases of mycotic thoracic aortic aneurysm using Valiant®. During device preparation before insertion, pyoktanin was flushed from the side port instead of saline containing heparin.

**Results:**

There were no operative deaths, recurrences of infection, or major complications. Two cases died from pneumonia and cancer; the other six cases were alive during the follow-up period.

**Conclusions:**

Pyoktanin-applied thoracic endovascular aortic repair for mycotic thoracic aortic aneurysm treatment is effective. However, the appropriate use of antibiotics and bundled therapy is necessary at present.

## Background

Although rare, mycotic thoracic aortic aneurysm (MTAA) is an extremely serious disease with a high rupture and high mortality rate. When only conservative therapy is provided, the mortality rate is believed to be over 80% (JCS Joint Working Group 2013). Therefore, treatment is generally based on surgery.

The basic surgical treatments include open aneurysm excision and aortic repair, debridement, and drainage coupled with the long-term administration of antibiotics. Thoracic endovascular aortic repair (TEVAR), a relative contraindication for MTAA, has previously been performed as a bridge therapy before open surgery or as an emergency surgery in cases such as ruptures. However, several cases have recently reported that TEVARs were performed for MTAA as elective surgery, with adequate outcomes and no recurrent infections (Isao et al. 2017). Nevertheless, there are still the problems of the infection focus remaining as a closure cavity and of postsurgical infection due to the device placed in the infection focus.

The Valiant® thoracic stent graft system (Medtronic, Santa Rosa, CA, USA) is our primary choice in our elective TEVAR, although it is also used in MTAA. Because the above-described problem of device infection remains, pyoktanin (methylrosanilide chloride) is applied to our graft prior to insertion to prevent infection. Thus far, this technique has been used in all MTAA cases in our institution, and all patients have survived at least 15 months after TEVAR, implying that pyoktanin renders a certain amount of protection against device infection. Further, pyoktanin is easily prepared and stored. Moreover, stock can be quickly made for urgent or emergent surgery, making it convenient for use. Here we report our clinical experience with pyoktanin-applied TEVAR for the treatment of MTAA, including its effects. To the best of our knowledge, such an experience has not yet been reported.

## Patients and methods

### Study design and background

This was a retrospective single-institution study. The use of pyoktanin-applied TEVAR for MTAA has been approved by the ethics committee at our institution.

Previous reports have dealt with how to flush rifampicin (RFP) into surgical graft in graft replacement as a means of treating MTAA before performing the replacement (JCS Joint Working Group 2013; Koshiko et al. 2002). However, an investigation of the literature revealed that there were no reports of pyoktanin being used in surgical graft or stent-graft and no reports on techniques wherein pyoktanin is infused among reports of surgeries performed using TEVAR as a curative treatment for MTAA.

As shown below, we use pyoktanin at our institution when surgical graft or stent-graft is used at the site of infection, as we have found that the sustained efficacy for RFP is poor when surgical graft or stent-graft is used for MTAA.

### Patient selection

Patients with MTAA who were admitted to our institution and for whom the decision was made to perform surgery underwent graft replacement that was infused with pyoktanin if it was determined that they could tolerate thoracotomy. TEVAR using stent grafts that were infused with pyoktanin was performed on patients who were determined unable to tolerate thoracotomy procedures, such as patients with numerous complications or a poor general condition. All patients meeting the above conditions were evaluated.

### Pyoktanin-applied TEVAR procedure

We performed pyoktanin-applied TEVAR for eight MTAA cases. The clinical information for the eight cases is listed in Table 1.

**Table 1:** Preoperative characteristics of patients undergoing TEVAR for MTAA

No	Age	Sex	DM	HT	HD	At admission					Pre-admission Abx	Rupture after admission
						Fever	Pain	WBC	CRP	Blood culture		
1	73	male		+			+	5700	1.88		+	
2	78	male		+			+	22,900	30.4		+	
3	70	female		+		+	+	10,500	8.71	+		+
4	82	male		+	+			9800	5.67		+	
5	85	male	+		+		+	12,500	10.79	+	+	
6	85	male	+		+	+		5900	5.59		+	
7	87	male	+	+			+	12,400	17.53	+		
8	60	male		+				12,700	16.1			

## Results

There were one female and seven male patients, with a mean age of 77.5 years. Three cases were complicated with diabetes mellitus, and three persons were hemodialysis patients. Major complaints included fever (*n* = 2), back pain (*n* = 5), and back pain associated with fever (*n* = 1). All patients presented with symptoms of infection in their blood test results at the time of their first visit to our hospital, including five patients who had previously received antibiotics at other hospitals. Three patients exhibited positive blood culture results at the time of hospitalization. No3 patient was detected *staphylococcus aureus*, No5 was detected *enterococcus faecalis* and No7 was detected *enterococcus faecium* from each blood cultures. The patients were administered antibiotics from the time of hospitalization and were treated to improve inflammation symptoms, which was monitored with regular computed tomography (CT) scans.

TEVAR was performed electively except for one emergency case because of an in-hospital rupture. The mean time from admission to TEVAR was 13.2 days (range: 7–18 days). All patients received surgery using pyoktanin-applied TEVAR under general anesthesia. All surgeries were successful with no complications. Following the surgery, patients continued receiving intravenous antibiotics until the white blood cell count fell within the normal range or C-reactive protein was ≤5. Thereafter, oral antimicrobial therapy was continued. Patients were discharged once it was confirmed that there were no recurrent signs of inflammation and no bacterial growth in their blood culture. Oral antimicrobial therapy was continued until the signs of inflammation were completely resolved at the patient’s visit. The mean time from surgery to discharge was 26.5 days (range: 12–66 days). The mean follow-up time from surgery was 28.4 months (range: 13–64 months) (Table 2).

**Table 2:** Surgical data, duration of antibiotic administration after operation, and outcome

No	Duration to ope. (d)	Hospital stay (d)	Antibiotic duration after ope.		Follow-up period (m)	Outcome
			IV (d)	Oral (m)		
1	12	12	5	2	64	alive
2	17	14	7	3	39	alive
3	7	28	19	6	30	death from pneumonia
4	10	14	10	3	29	alive
5	14	66	35	4	19	alive
6	15	29	21	6	18	alive
7	13	28	12	6	15	death from pneumonia
8	18	21	11	3	13	alive

### Case study for patient #6

**Fig. 1:**
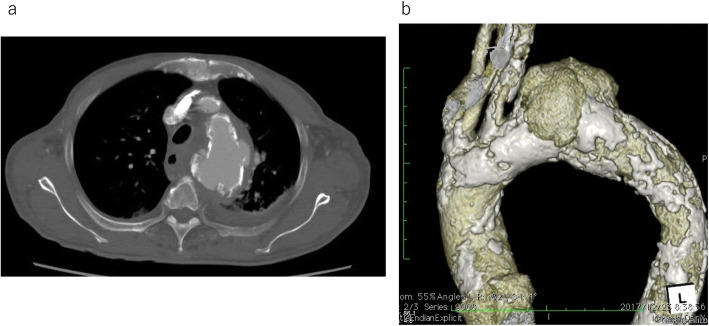
**a** Multiloculated cyst-like aneurysm without pleural effusion. **b** The aneurysm is located at the distal arch, with a maximum diameter of 74 mm

Case #6 was an 85-year-old male who currently visits another hospital for hemodialysis treatment. An unknown fever was observed, with inflammation signs in a blood test at the previous hospital. Following hemodialysis, treatment with intravenous antibiotics was initiated, but there was no improvement. *Escherichia coli* were detected in the patient’s blood culture, and he was referred to our institution. His CT scan at the time of hospitalization showed a multiloculated cyst-like distal arch aneurysm, with a maximum diameter of 74 mm (Fig. 1a, b). Although the imaging findings suggested an impending rupture, the patient noticed no symptoms. Considering that the signs of inflammation were evident, meropenem (0.5 g/d) and daptomycin (350 mg/d) were initiated. After the administration of these antibiotics, the signs of inflammation improved, and the fever was reduced. However, the signs of inflammation were not completely resolved, and a CT exam on day 13 revealed a further enlargement of the aneurysm, which led to urgent TEVAR surgery.

TEVAR was performed with Valiant® VAMC3228C150TJ under general anesthesia. Typically, saline containing heparin is flushed from the side port as a pretreatment prior to device insertion. In this case, however, pyoktanin was used instead of saline containing heparin. Then, the device was deployed as per the usual procedure.

**Fig. 2:**
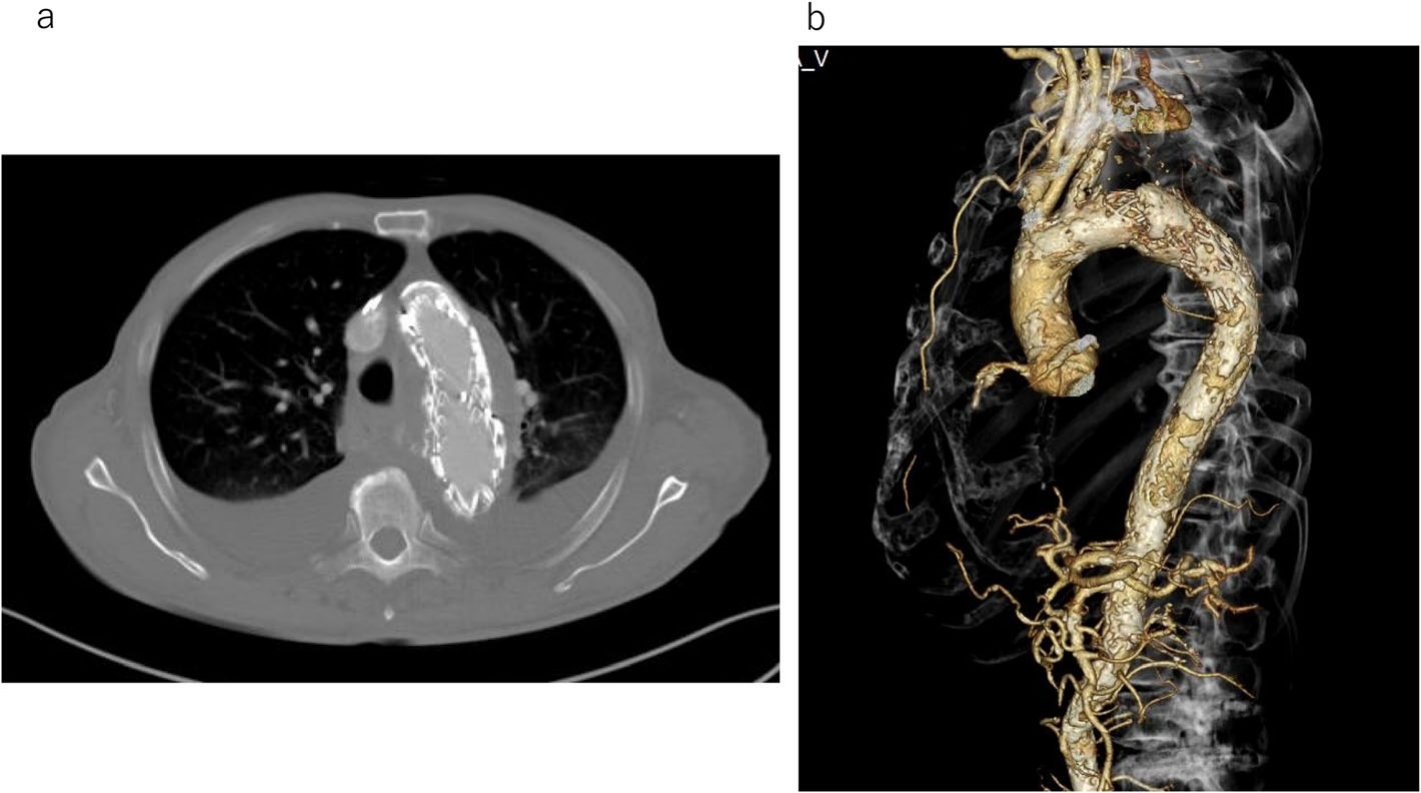
**a** Postoperative enhanced CT. No leakage is detected, and the aneurysm is shrinking in size. **b** No retrograde type A aortic dissection is detected

Following the surgery, the administration of meropenem and daptomycin was continued. Daptomycin was discontinued on postsurgical day 10, and meropenem was discontinued on postsurgical day 29, based on the signs of inflammation and blood culture results. Oral antimicrobial therapy then commenced, and after confirming that there were no recurrent signs of inflammation, the patient was discharged on postsurgical day 41. A postoperative enhanced CT showed no leakage to the aneurysm (Fig. 2a, b). Currently, at 18 months post-surgery, the patient visits our hospital regularly and shows no recurrent signs of inflammation. Furthermore, the imaging exam shows a reduction in the aneurysm size.

## Discussion

Infected aneurysm, a disease reported by Osler for the first time in 1885, is a general term for describing aneurysms caused by infection and infection-affected pre-existing aneurysms (JCS Joint Working Group 2013). It is a relatively rare disease, accounting for only 0.5%–1.3% of all aortic aneurysms (Muller et al. 2001). The mortality rate of 23.5%–37% is significantly high compared to its non-infectious counterpart. No treatment has been established so far. The only current approach to treatment is planning therapy for the individual patient while taking into account the condition of the aneurysm and the patient’s background (Muller et al. 2001).

Causes include infected endocarditis, bacterial arteritis, infection of preexisting aortic aneurysms, and traumatic or iatrogenic factors. Although there is research reporting that *Salmonella, E. coli*, *Staphylococcus*, *Streptococcus*, etc., were detected as the causative bacteria and > 70% of cases had positive culture results, other reports show that > 50% of the cases had negative culture results. It should be noted that negative results in the blood culture do not negate the possibility of MTAA (Isao et al. 2017).

Before treating the MTAA, a sufficient and required dose of antibiotics should first be administered to preferably suppress and infection prior to surgery. Because the mortality rate is reported to be 54.5%–96% if treated with antibiotics alone, the presurgical use of antibiotics, wherever applicable, has been recommended as a standard procedure (Hsu et al. 2004; Forbes and Harding 2006). Previously, the surgical procedure included the removal of infected tissues by excising the aneurysm, aortic repair, and omental wrapping (Kan et al. 2007; Satoshi et al. 2013). However, although the outcome varied depending on the preoperative systemic conditions, many cases involved poor preoperative conditions with extensive surgical invasion, and the in-hospital and one-year mortality rates were reported to be 11% and 25%, respectively, which cannot be considered a satisfactory outcome (Hsu et al. 2004). Recently, indications for TEVAR, which is a minimally invasive endovascular treatment, have been increasing for atherosclerotic thoracic aortic aneurysms. Since the introduction of aortic stent grafts for infected abdominal aortic aneurysms by Semba et al. for the first time in 1998, better outcomes in combination with appropriate antibiotic therapy have been reported for infected aortic aneurysms, and this is considered an effective alternative to the conventional strategy (Kan et al. 2010).

TEVAR has been, in principle, considered a relative contraindication for infected aortic aneurysms from the viewpoint of the insertion of an artificial device into the infected site and has been performed in cases where invasion by surgical revascularization was impractical owing to a poor systemic condition or as an emergency measure before open surgery (Forbes and Harding 2006). However, because MTAAs are in saccular aneurysmal changes usually, TEVAR is easier to manipulate and quickly stops hemorrhages in rupture cases. Thus, many believe that TEVAR is useful for ruptured cases associated with infection (Bell et al. 2003). However, it should be noted that the postsurgical course may vary, and postsurgical administrations of antibiotics to prevent the prolongation and recurrence of infection, along with careful follow-ups, are necessary.

Kan et al. investigated 48 cases where stent grafts were used for infected aortic aneurysms and reported surgical deaths in 10.4% (five cases), with a one-year survival rate of 94% (infection- treated group) compared to 39% (infection-non-treated group) (Kan et al. 2010). Another study reported a 30-day mortality rate of 5.6% (Kan et al. 2007). In Europe, TEVAR was performed in 130 cases of infected aortic aneurysms at 16 centers, reporting additional surgery by replacing the aneurysm with synthetic graft in 5% of cases, with a one-year survival rate of 91% and an infection-related mortality rate of 19% (Sörelius et al. 2014). Other reports suggest the validity of stent grafting combined with surgical debridement (Prokakis et al. 2008). There are also reports of cases where antibacterial agent-treated grafts were used.

In surgery for MTAA, expanded polytetrafluoroethylene (ePTFE) grafts and RFP-soaked ePTFE grafts have been used, and there are reports on such usage. Narasimhan et al. reported that ePTFE graft did not allow methicillin-resistant *Staphylococcus aureus* (MRSA) and *E. coli* to pass through the vessel wall, even when their biofilm was formed on the vessel surface (Narasimhan et al. 2010). As far as our research is concerned, there were no reports of increased postsurgical infections between the use of ePTFE graft and non ePTFE grafts (Taichi et al. 2016; Hai Lei and Yui Che 2018). This suggests that there will be no differences among devices used in TEVAR for MTAA, although the results are not conclusive because the anti-infection properties of these devices have not been investigated.

Studies have reported on the use of grafts treated with RFP, an antimicrobial agent. Because RFP strongly binds to gelatin and is effective across a broad spectrum, it is used in gelatin-coated synthetic vessels. A decrease in the mortality rate and the recurrent infection rate by REP-soaked grafts has been reported. However, in Japan, the RFP solution is not commercially available and is currently prepared in-hospital at the time of use. Although the RFP solution is available overseas, in Japan, the RFP solution must be prepared from powders. Dissolving RFP powders is an awkward and rather laborious task. Another problem is its short lifetime in solution; consequently, the effectiveness of RFP is lost during long-term storage. In addition, a report suggests that while RFP is effective on weak bacteria, such as *Staphylococcus epidermidis*, its effects on MRSA are unknown, because MRSA can be tolerated immediately(Koshiko et al. 2002).

At our institution, to treat mediastinitis after graft replacement, the cleaning of the lesion and removing of infected tissues have been extensively performed before omental wrapping as much as possible in addition to administering antibiotics. Pyoktanin has been applied to the artificial vessel at the time of cleaning the lesion to control the local infection. Pyoktanin has been used as an antibacterial agent for grafts at our institution because in addition to the fact that we have not adopted the use of RFP, RFP requires time-consuming preparation as described above, and the cost of pyoktanin is lower than that of RFP.

Pyoktanin (methylrosanilide chloride) is a synthetic dye that belongs to the triphenylmethane family, and it has an antiseptic effect. It was first synthesized in the 1860s and used for medical treatment by Stilling from 1890 onward (Akira et al. 2016). Pyoktanin is considered to have an antibacterial effect against gram-positive bacteria, particularly staphylococci and diphtheria bacilli, and is widely used for skin ulcers and decubitus infections (Alexander and Jack 2013). Its primary effect is the inhibition of cell wall and glutamine synthesis at sites different from penicillin. Even in MRSA, Pyoktanin is very effective antiseptic effect (Akira et al. 2015). Salmonella are gram-negative anaerobic bacteria which is one of the main bacteria with mycotic aneurysm. Although there is not examined Salmonella, there is research that pyoktanin is effective against Gram-negative (Ichino et al. 2015), so we thought that pyoctanin might be effective against MTAA, so we decided to use it for MTAA at our institution.

Pyoktanin is recommended for administration at a 0.1% concentration and provides a relatively stable efficacy against MRSA without causing tissue damage. Maley et al. reported that at a concentration of 0.1%, pyoktanin has almost no side effects and can be safely used (Maley and Arbiser 2013). Disinfectant solutions, such as RFP and povidone-iodine, quickly weaken and lose their potency in the presence of albumin and in regions with a large amount of exudate, whereas pyoktanin can be expected to still render its effects, even in the presence of albumin, and it is known for its persistent and stable antibacterial effects that can last 24 h in regions with a large amount of exudate (Jun and Yoshihiro 1992).

In our institution, a 0.1% formulation of pyoktanin is prepared by dissolving the powders in a solvent under sterile conditions. It can be readily prepared, even for emergency surgery, because the time required for preparation is only around 1 h, including the time for sterilizing the solvent. When used in surgery, the solvent is tested in culture prior to use to confirm its sterility.

In our institution, 151 cases of TEVAR surgery, including MTAA cases, aortic dissections, and aortic ruptures, were performed from April 2014 to July 2018. There were four cases of death (2.6%) (including two rupture cases), two cases of complications with cerebral infarction (1.3%), and two cases of complications with spinal infarction (1.3%). Valiant® was used in 112 (74%) of the cases. Although there are many commercial products available for TEVAR and their superiority/inferiority cannot be easily compared, we believe that Valiant® is effective in various ways, such as the ease of handling, precise deployment at the placement site, tracking performance in the aorta, and strength of the radial force. MTAA was performed with Valiant® without problems in all cases.

There is no academic paper that describes drug penetration methods when treating MTAA with TEVAR for the purpose of clinical practice. However, from personal experience, when using the RFP solution, the stent was deployed once to allow RFP to penetrate into the graft and then stored into the catheter again to perform the insertion for delivery. Furthermore, re-insertion is troublesome, and there are potential risks of damage to the graft at the time of re-insertion and problems at the time of deployment that are due to handling during re-insertion. At our institution, we permeate the drug into the graft by injecting pyoktanin via a side port for evacuating the air. This is easily performed without requiring the procedures mentioned above. However, we do not know whether the pyoktanin is uniformly and securely applied to the device. Thus, the actual extent of permeation of the pyoktanin application was examined by injection in vitro.

**Fig. 3:**
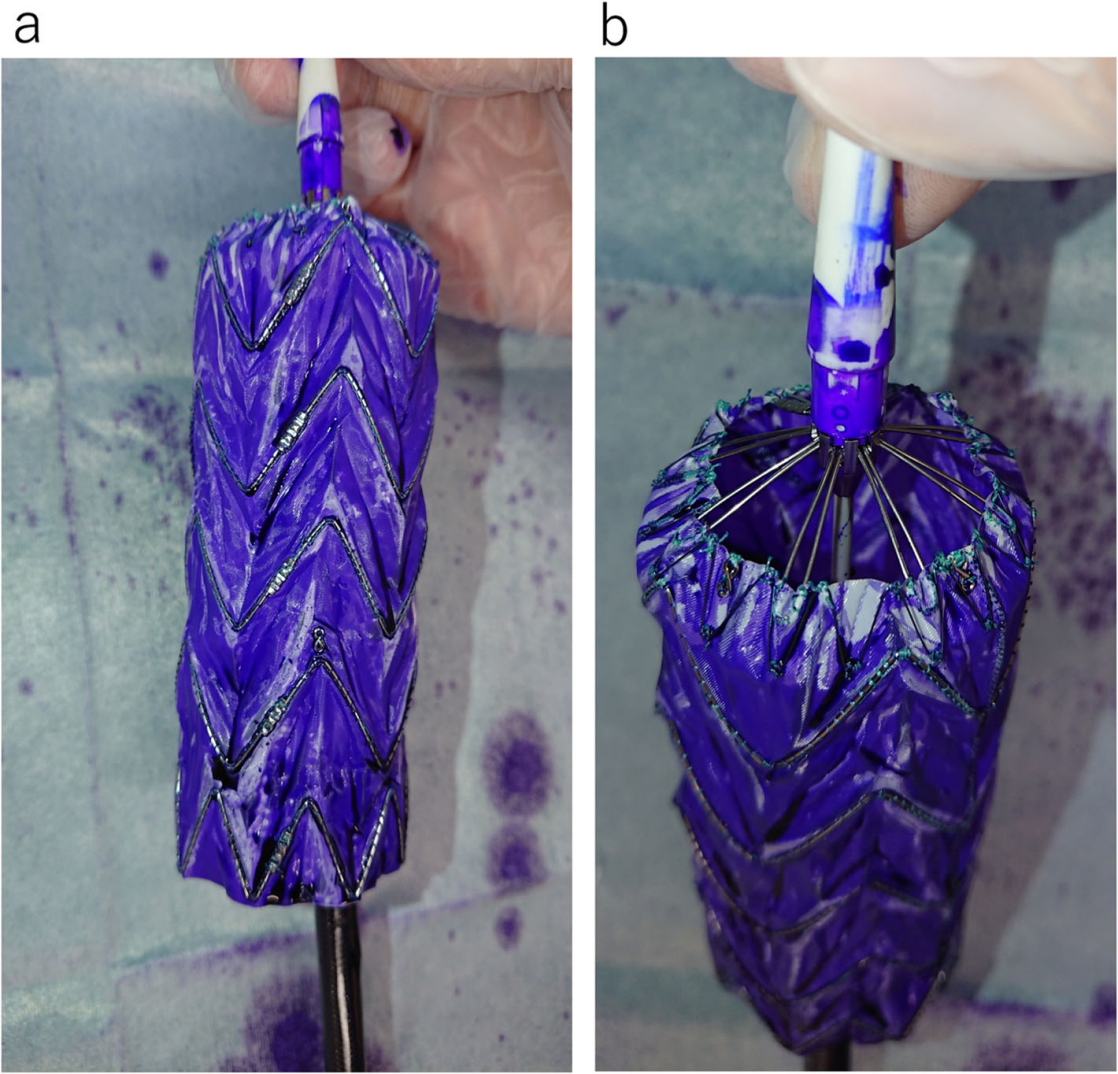
**a** soaked Valiant®-soaked pyoktanin after the injection of 40 mL of pyoktanin. **b** The wrinkled portion of Valiant® is not being dyed, but > 98% of the surface area is penetrated

**Fig. 4:**
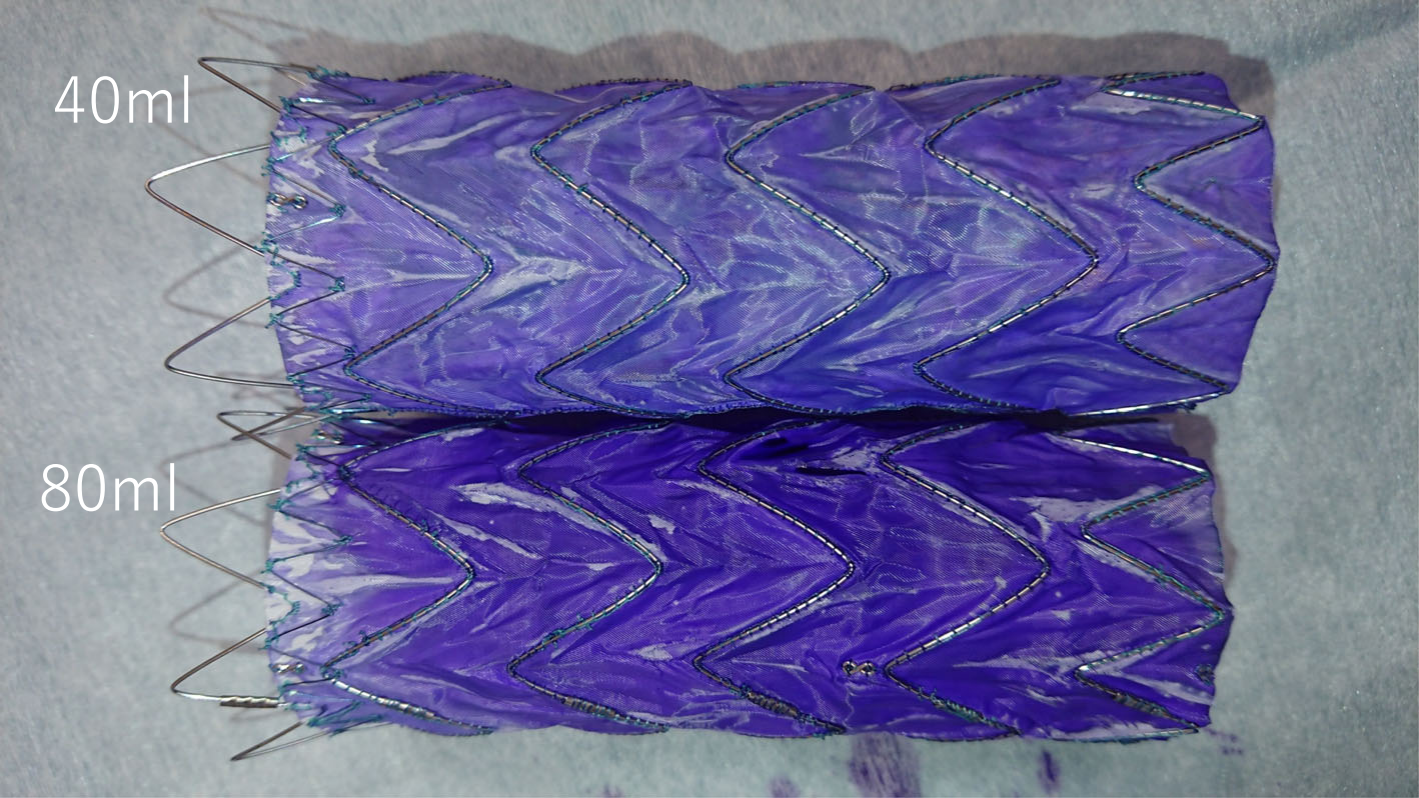
There was no difference between the amount of stained surface area between the 40-mL flush and 80-mL flush of pyoktanin

**Fig. 5:**
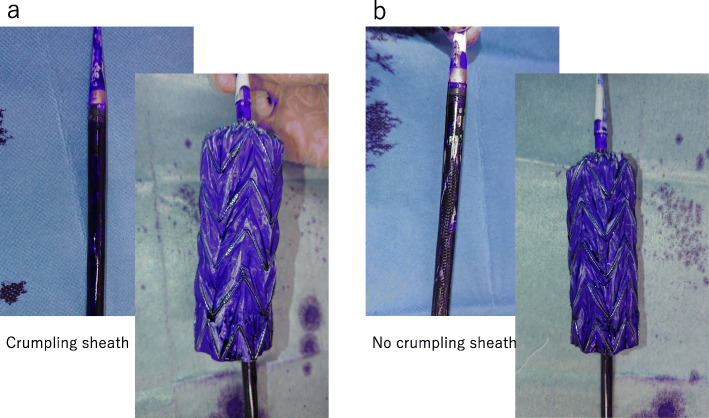
Comparison of the dyeing condition on the device between crumpling the outer sheath (**a**) and not crumpling the outer sheath (**b**). No difference was noted between the two methods

Flushing with 40-mL saline via a side port is recommended by the manufacturer. There is no difference in viscosity between the saline and pyoktanin solution; thus, we attempted flushing 40-mL pyoktanin via a side port. Developments after a while confirmed that pyoktanin had penetrated most areas (≥98% in terms of surface area ratio), except for the wrinkled part of the Valiant® graft (Fig. 3a, b). Next, 80-mL pyoktanin was flushed, and the device was removed. The graft was more intensely stained with pyoktanin, although there was no difference in the degree of penetration in terms of surface area compared to the 40-mL flush (Fig. 4). Crumpling the outer sheath at the time of pyoktanin flush did not change the degree of penetration in terms of surface area (Fig. 5a, b). In both cases, however, pyoktanin penetrated the area over time into areas where pyoktanin had been absent. This same phenomenon is hypothesis to also occur in the human body.

While there is a correlation between the pyoktanin concentration and the antibacterial activity, there is no correlation between the pyoktanin dose and its antibacterial effect. Although an 80-mL flush can penetrate the device at a stronger concentration, this fact suggests that the 40-mL flush can render the same antibacterial effect as an 80-mL flush. Therefore, there will be no problem with using pyoktanin for MTAA at a flush volume of 40-mL, which is equivalent to the same amount of saline recommended by the manufacturer for normal operation.

No patients in this study exhibited any recurrent signs of infection. However, it is possible that in cases of strong infection, concentrated infusions of pyoktanin alone might be insufficient. Because this treatment requires that an artificial object be inserted at the site of infection, physicians must constantly check for any signs of further infection postoperatively.

Our study had some limitations. First, the sample size was small, and our results need to be confirmed across a larger population. Second, this was a single-institution study, and future investigations should be performed across multiple institutions. And last, there are not able to compare the use group and non-use group of pyoktanin. This would entail the use of a specific standardized protocol for all institutions, so that the results are comparable.

Our results suggest that pyoktanin-applied TEVAR for MTAA treatment can be easily implemented and is an effective treatment. Although the appropriate use of antibiotics and bundled therapy are necessary at the present time, pyoktanin-applied TEVAR can serve as an effective treatment for MTAA. Further investigation with more clinical cases is planned at our institution.

## Conclusions

MTAA is high mortality disease and TEVAR for MTAA as radical therapy is still controversially method. But pyoktanin-applied TEVAR for MTAA is effective we thought, and the appropriate use of antibiotics and bundled therapy is necessary at present.

## Data Availability

Not applicable.

## Abbreviations

CT: Computed tomography
MRSA: Methicillin-resistant *Staphylococcus aureus*
MTAA: Mycotic thoracic aortic aneurysm
TEVAR: Thoracic endovascular aortic repair
RFP: Rifampicin
